# Comparative Transcriptome Analysis of CCCH Family in Roles of Flower Opening and Abiotic Stress in *Osmanthus fragrans*

**DOI:** 10.3390/ijms232315363

**Published:** 2022-12-06

**Authors:** Yong Ye, Shanshan Cao, Lixiao Shen, Yiguang Wang, Shiwei Zhong, Liyuan Yang, Zheng Xiao, Qiu Fang, Hongbo Zhao, Bin Dong

**Affiliations:** 1 School of Landscape Architecture, Zhejiang Agriculture and Forestry University, Hangzhou 311300, China; 2 Zhejiang Provincial Key Laboratory of Germplasm Innovation and Utilization for Garden Plants, Hangzhou 311300, China; 3 Key Laboratory of National Forestry and Grassland Administration on Germplasm Innovation and Utilization for Southern Garden Plants, Hangzhou 311300, China

**Keywords:** zinc finger family, phylogenetic analysis, expression pattern, *Osmanthus fragrans*

## Abstract

CCCH is a zinc finger family with a typical CCCH-type motif which performs a variety of roles in plant growth and development and responses to environmental stressors. However, the information about this family has not been reported for *Osmanthus fragrans*. In this study, a total of 66 *CCCH* predicted genes were identified from the *O. fragrans* genome, the majority of which had multiple CCCH motifs. The 66 *OfCCCHs* were found to be unevenly distributed on 21 chromosomes and were clustered into nine groups based on their phylogenetic analysis. In each group, the gene structure and domain makeup were comparatively conserved. The expression profiles of the OfCCCH genes were examined in various tissues, the flower-opening processes, and under various abiotic stresses using transcriptome sequencing and qRT-PCR (quantitative real-time PCR). The results demonstrated the widespread expression of *OfCCCHs* in various tissues, the differential expression of 22 *OfCCCHs* during flower-opening stages, and the identification of 4, 5, and 13 *OfCCCH*s after ABA, salt, and drought stress treatment, respectively. Furthermore, characterization of the representative OfCCCHs (OfCCCH8, 23, 27, and 36) revealed that they were all localized in the nucleus and that the majority of them had transcriptional activation in the yeast system. Our research offers the first thorough examination of the *OfCCCH* family and lays the groundwork for future investigations regarding the functions of CCCH genes in *O. fragrans*.

## 1. Introduction

Zinc finger proteins are the largest TF (transcription factor) families in eukaryotes and consist of a sequence of one or more cysteine residues with a histidine residue [1,2]. Based on the number and spacing between the conserved cysteines and histidine residues, zinc-finger TFs can be categorized into nine types, including C2H2, CCCH, C3HC4, C4HC3, C2HC5, C2HC, C4, C6, and C8 [3]. Among them, the CCCH zinc-finger TFs as a specific zinc-finger family was first found by Wang et al. in *Arabidopsis* [2], which contains a typical CCCH-type zinc-finger motif with three conserved cysteines and one histidine residue, accounting for approximately 0.8% of all the zinc finger proteins [4]. In most species, various members of the CCCH zinc-finger family have been identified by genome-wide analysis, including 68 in *Arabidopsis* [2], 91 in *Populus trichocarpa* [5], 49 in *Dimocarpus longan* [6], 88 in wheat [7], and 155 in *Brassica napus* [8].

Many studies have reported that CCCHs play crucial roles in plant growth and development and adaptive processes. In *Arabidopsis*, a tandem zinc-finger protein AtTZF3 mediates seed germination under salt treatment as a negative regulator [9]. *HUA1* contains CCCH-type zinc finger motifs, of which biological function is involved in stamen/carpel identities and floral determinacy by RNA-binding [10]. Meanwhile, *HUA1* also regulates plant size, and *hua1* mutant lines are smaller and shorter than WT (wild type) plants [10]. In rice (*Oryza sativa* L.), CCCH protein SAW1 (swollen anther wall 1) controls anther development by regulation of gibberellin homeostasis [11]. In addition, CCCH type gene *IIP4* (*ILA1-interacting protein 4*) is confirmed in relevant transgenic plants by Zhang et al. [12], and the gene can regulate secondary wall formation and control the mechanical strength of rice. In *Cucumis sativus*, CsSEF1 (Somatic Embryogenesis Zinc Finger 1) is required for somatic embryogenesis and root development [13]. Furthermore, most research shows that CCCH proteins are involved in hormone signaling and influence plant growth and development. In *Arabidopsis*, *AtTZF4*, *5*, and *6* are only expressed in seeds, and the three CCCH-type genes involved in ABA-(abscisic acid) and GA-(gibberellic acid) mediate the regulation of seed development [14]. PvCCCH69 is a repressor of leaf senescence as it suppresses the abscisic acid-signaling pathway in switchgrass [15]. In addition, accumulating evidence indicates that a number of *CCCH* genes participate in plant abiotic stresses and defense responses. For example, the non-tandem CCCH type zinc finger protein IbCCCH18 enhances abiotic stress tolerance in sweet potato (*Ipomoea batatas*) [16]. The expression of *OsTZF5* (CCCH-tandem zinc finger protein 5) increases due to abscisic acid and dehydration stress, and the overexpression of *OsTZF5* promotes rice drought tolerance [17]. In Moso bamboo (*Phyllostachys edulis*), *PeCCCH74* enhances drought tolerance and improves the survival rate of *Arabidopsis* under drought stress treatment [18]. In Switchgrass, plant chilling and freezing tolerance can be significantly improved in the *PvCCCH72* transgenic lines through the ABA-mediated pathway [19].

*O. fragrans*, one of the top ten traditional flowers in China, is an excellent ornamental and practical garden tree that integrates greening, beautification, and fragrance. This ornamental plants have been widely cultivated in Asia due to its unique fragrance, aesthetic and cultural values. However, CCCH genes have not been identified in *O. fragrans*, and their functions are poorly understood in *O. fragrans*. In this study, we first performed a genome-wide identification of *CCCH* family members in the *O. fragrans* genome. Their chromosomal locations, phylogenetic relationships, gene structural, domain, and CRE (cis-regulatory element) features were then characterized. Finally, the profiles of expression of the *CCCHs* were explored during flower opening and abiotic stress. In summary, this work provides new insights into the *CCCH* genes involved in the regulation of flower opening and abiotic stress tolerance, which improves knowledge regarding the diverse functions and features of *CCCHs* in the breeding of *O. fragrans*.

## 2. Results

### 2.1. Genome-Wide Identification of the OfCCCHs

**Figure 1:**
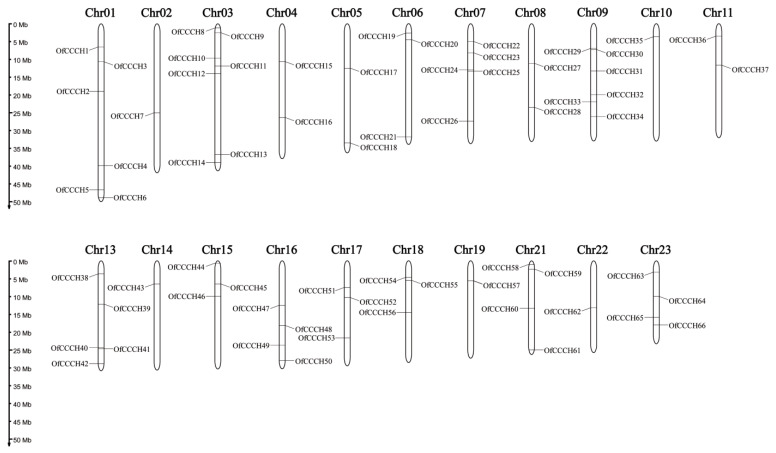
Chromosomal distribution of 66 *OfCCCH* genes on 21 *Osmanthus fragrans* chromosomes.

A total of 66 *CCCHs* that contained a typical *CCCH* domain were identified from the *O. fragrans* genome and were named *OfCCCH1*-*66*. The characterizations of the 66 *OfCCCH* genes are shown in Table S1, including the number of amino acids (length), molecular weights, pI (isoelectric points), and the physical location. The detailed information indicated that the length of the 66 *OfCCCH* proteins varied and ranged from 127 amino acids (*OfCCCH37*) to 1195 amino acids (*OfCCCH46*). In addition, the pI of the 66 *OfCCCHs* ranged from 4.54 (*OfCCCH39*) to 9.47 (*OfCCCH38*). All the *OfCCCH* genes were mapped on 21 chromosomes, and the highest proportion was located on chromosome 3 (7 members) (Figure 1).

### 2.2. Phylogenetic Analysis, Gene Duplication, and Synteny Analysis

**Figure 2:**
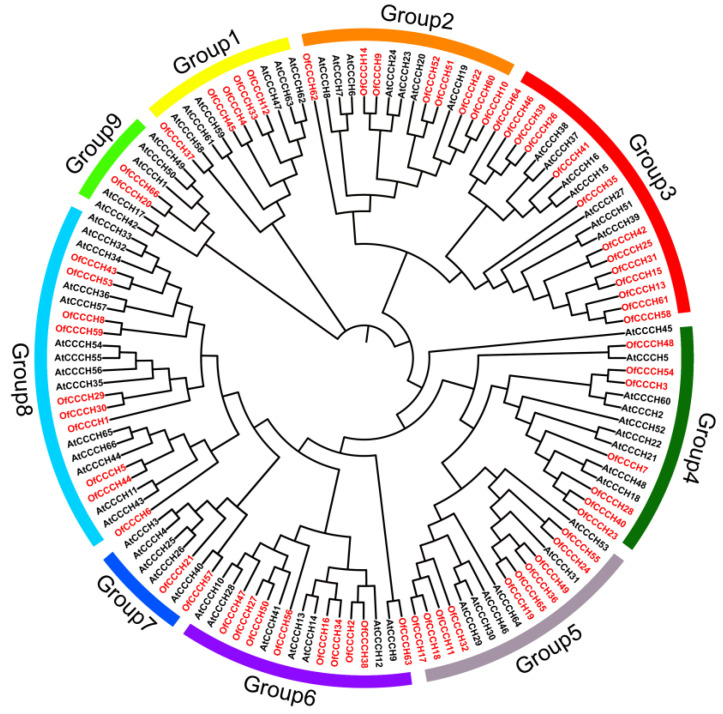
Phylogenic relationship of the CCCHs in *Osmanthus fragrans* and *Arabidopsis thaliana*. The red font represents CCCHs from *Osmanthus fragrans* and the black font represents CCCHs from *Arabidopsis thaliana*.

**Figure 3:**
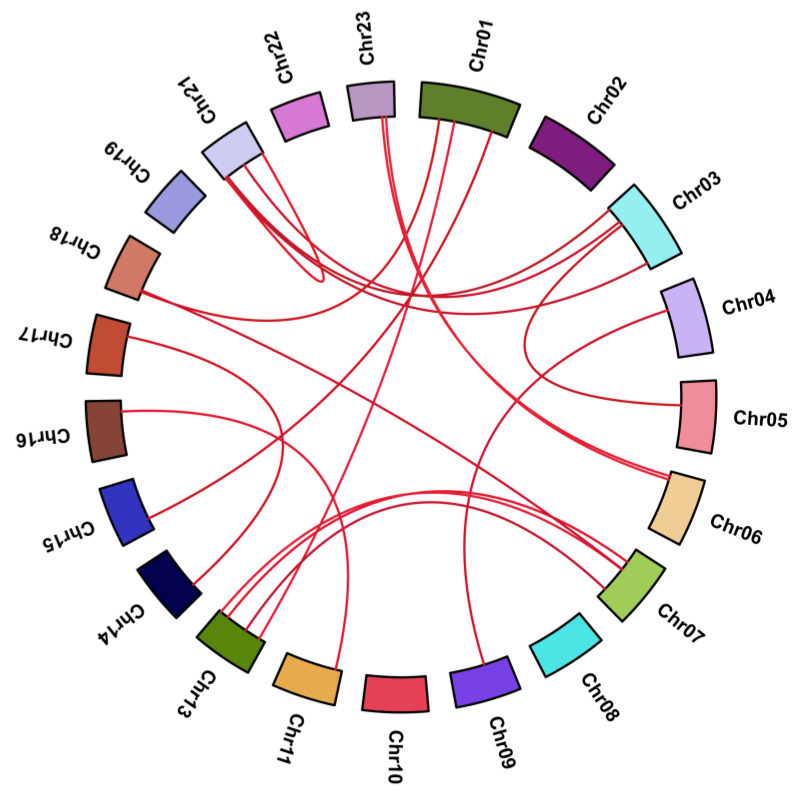
Synteny analysis of 66 *CCCHs* in *Osmanthus fragrans*. The red lines suggest duplicated *CCCH* gene pairs in the *O. fragrans* genome.

Based on phylogenetic analysis, the CCCH proteins of *Arabidopsis* and *O. fragrans* were divided into nine groups (Figure 2). A total of 114 of 132 CCCHs fell into one branch and were further divided into two subgroups. The first included 79 CCCHs, and the second consisted of 35 CCCHs. In contrast, the other two branches separated from the previous branch, including 7 and 11 CCCHs, respectively, demonstrating their phylogenic dissociation from the other one. Seventeen segmentally duplicated genes were identified, and no tandem duplicated gene was found. Segmental duplication events were suggested to be the only cause of *OfCCCH* gene expansion. Remarkably, chromosome 21 had the largest number of segmentally duplicated gene pairs (Figure 3, Table 1). Among the 17 duplicated gene pairs, all of them evolved under purifying selection (Ka/Ks < 1) (Table 1). The time of divergence of the *OfCCCHs* implied that the duplication events occurred 37.57 million years ago (Mya) and continued up until 0.37 Mya, while the most frequent gene duplication events occured 0–10 Mya (Table 1).

### 2.3. Gene Structure, Domain, and CREs Analysis

**Figure 4:**
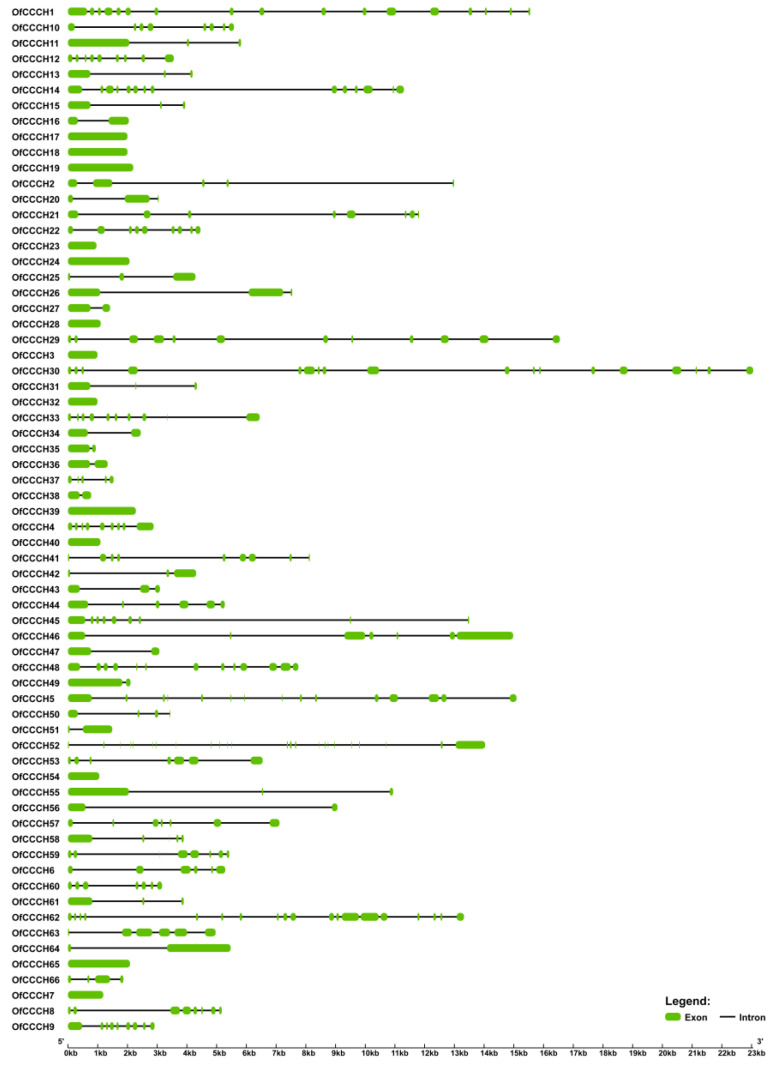
Gene structure analysis of *OfCCCH* genes. The black lines and green rectangles represent intron and exon, respectively.

**Figure 5:**
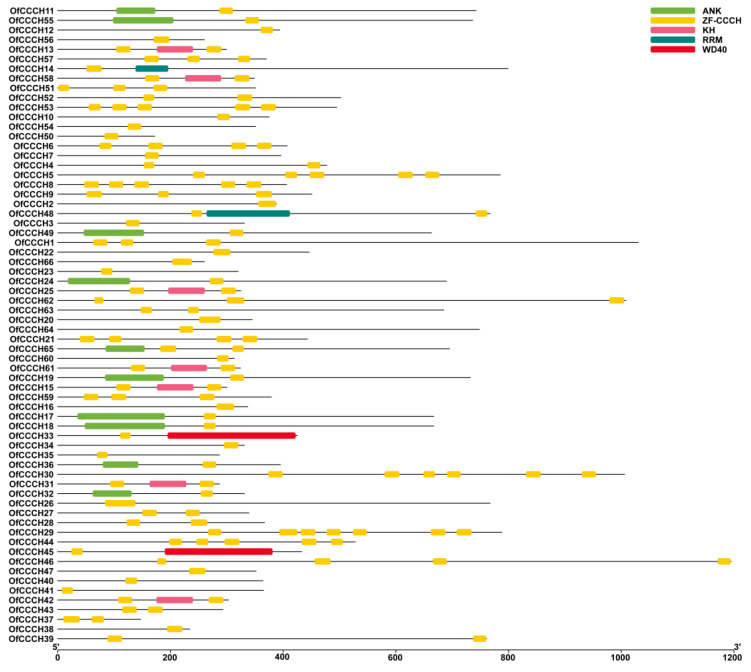
Domain analysis of OfCCCH proteins.

**Figure 6:**
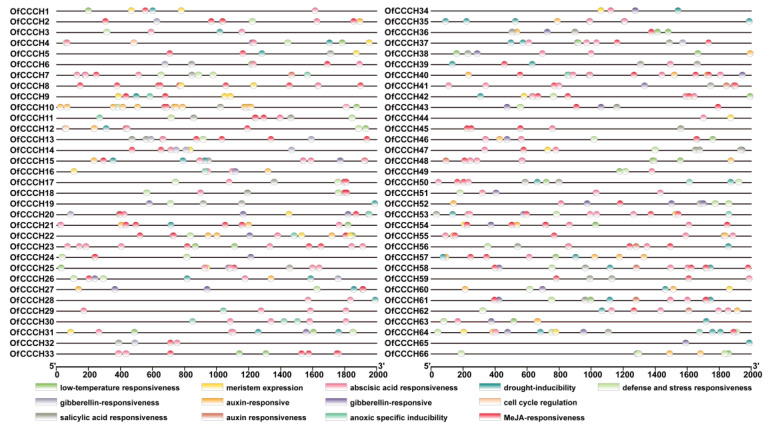
CREs (*Cis*-regulatory elements) analysis of the promoters of *OfCCCH* genes. Different CREs are represented by different colored ellipses.

To better understand the gene structure of *OfCCCHs*, the intron and exon were identified, and the number of exons ranged from 1 to 24 (Figure 4). In addition, a total of five types of motifs were found in the *OfCCCH* family, including ZF-CCCH, ANK, KH, RRM, and WD40 (Figure 5). Forty-five *OfCCCH* genes contained one motif, and the rest of the 21 *OfCCCH* genes possessed two type motifs. For the CREs analysis, a total of 13 CREs were identified in the promoters of 66 *OfCCCHs*, of which many were in response to hormones such as ABA, MeJA (methyl jasmonate), GA, auxin, and SA (salicylic acid) (Figure 6). It was determined that 56 of the *OfCCCH* promoters had ABA-responsive elements, which represented the largest proportion of all *OfCCCH* promoters, followed by the MeJA-responsive elements (50), the gibberellin-responsive elements (35), the auxin-responsive elements (35), and the salicylic acid-responsive elements (35), but none of these elements were shared by all *OfCCCH* promoters (Figure 6).

### 2.4. Transcriptome Analysis of OfCCCH Genes in Different Tissues

**Figure 7:**
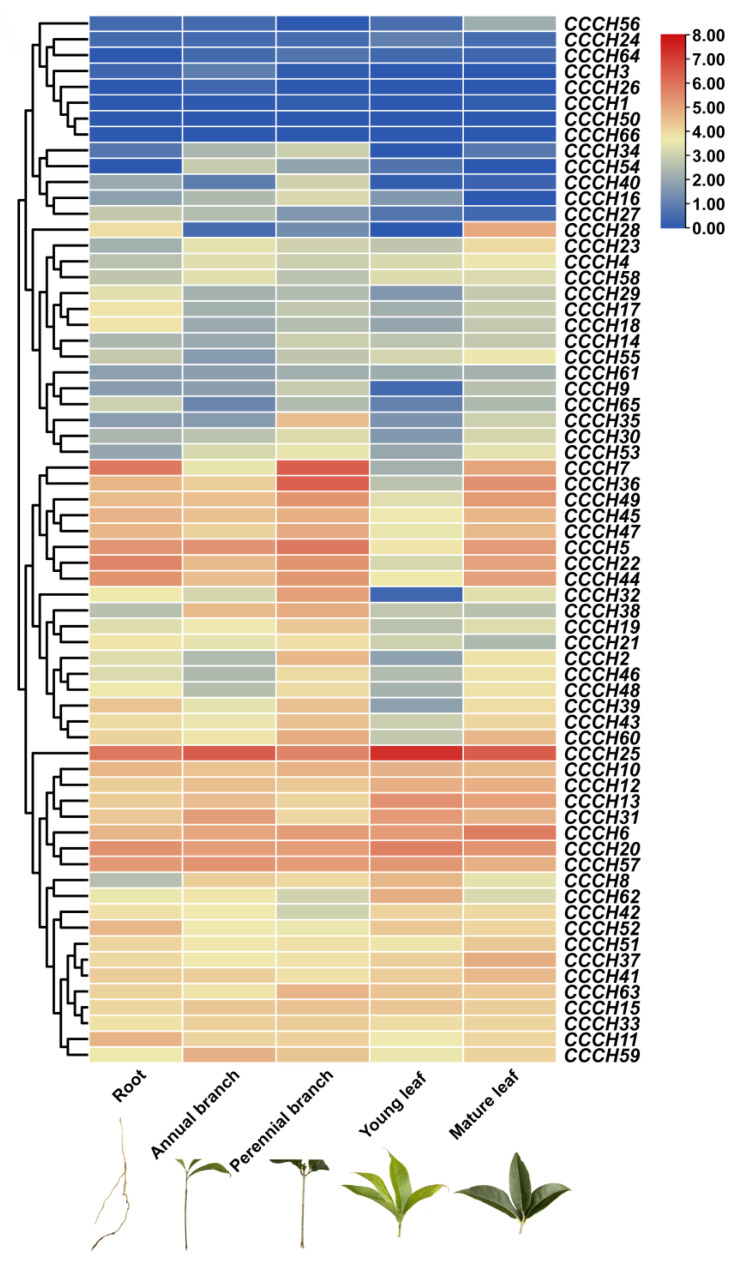
Expression profiles of *OfCCCH* genes in different tissues of *Osmanthus fragrans*. The color scale of the dendrogram represents FPKM values. Red and blue colors exhibit higher levels and lower values, respectively.

Transcriptome analysis was employed to explore the level of transcription of the *OfCCCH* genes in different tissues (including root, annual branch, perennial branch, young leaf, and mature leaf). As shown in Figure 7, most *OfCCCH* genes were widely expressed in different tissues; remarkably, high expression of all tissues was observed for *OfCCCH6*, *20*, *25*, and *57*. Apparent tissue-specific expression was observed for some *OfCCCH* genes (Figure 7). For example, *OfCCCH3* was predominantly expressed in the annual branch. The expression of five *OfCCCHs* (*OfCCCH7*, *32*, *35*, *36*, and *40*) was overrepresented in the perennial branch. *OfCCCH28* and *OfCCCH56* were primarily expressed in the mature leaf.

### 2.5. Transcriptome and qRT-PCR Analysis of OfCCCH Genes during the Flower Opening Processes

**Figure 8:**
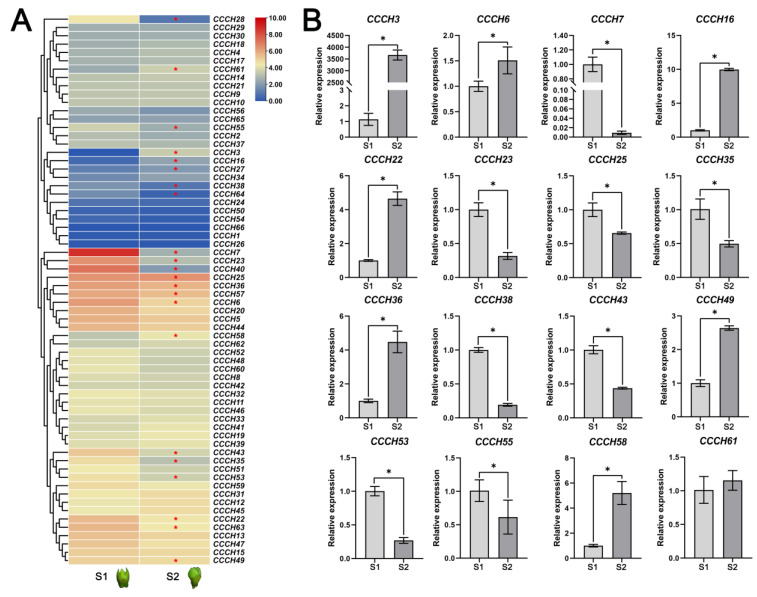
Expression profiles of *OfCCCH* genes during flower opening processes. (**A**) The expression values of the 66 *OfCCCHs* at S1 and S2 stages. The red asterisk represents differently expressed genes. (**B**) Expression profiles of *OfCCCH* DEGs by qRT-PCR. The black asterisk represents a significant difference (one-way ANOVA test), * represents a *p* value < 0.05.

Transcriptome was employed and the differently expressed genes were further analyzed to explore the roles of *OfCCCH* genes in the process of *O. fragrans* flower opening. Compared with the S1 stage of flower opening, a total of 22 *OfCCCHs* exhibited significantly differential expression in the S2 stage, including 9 upregulated *OfCCCH* genes (*OfCCCH3*, *6*, *16*, *27*, *36*, *49*, *57*, *58*, and *61*), and 13 downregulated *OfCCCH* genes (*OfCCCH*7, *22*, *23*, *25*, *28*, *35*, *38*, *40*, *43*, *53*, *55*, *63*, and *64*) (Figure 8A, Table S2). Subsequently, 16 DEGs (*OfCCCH3*, *6*, *7*, *16*, *22*, *23*, *25*, *35*, *36*, *38*, *43*, *49*, *53*, *55*, *58*, and *61*) of interest were selected among the 22 DEGs, and their expression patterns were detected in the S1 and S2 stages by qRT-PCR. As shown in Figure 8B, the qRT-PCR results were consistent with the expression trend of transcriptome. The expression of *OfCCCH3*, *6*, *16*, *22*, *36*, *49*, *58*, and *61* significantly increased from the S1 stage to the S2 stage, while *OfCCCH7*, *23*, *25*, *35*, *38*, *43*, *53*, and *55* significantly decreased from the S1 stage to the S2 stage (Figure 8B).

### 2.6. Transcriptome and qRT-PCR Analysis of OfCCCH Genes in Response to Abiotic Stress

**Figure 9:**
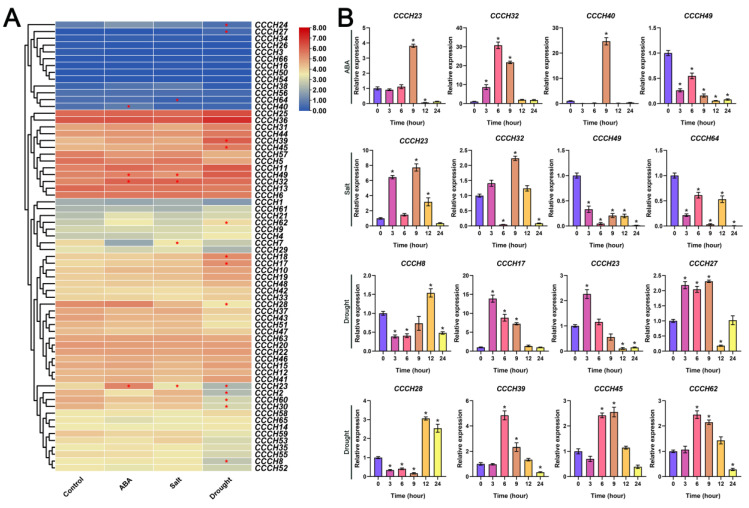
Expression profiles of *OfCCCH* genes under different abiotic stress treatments. (**A**) The expression values of the 66 *OfCCCHs* under ABA, salt, and drought stress. The red asterisk represents differently expressed genes. (**B**) Expression validation of *OfCCCH* DEGs at 0, 3, 6, 9, 12, and 24 h after stress treatments. The black asterisk represents a significant difference (one-way ANOVA test), * represents a *p* value < 0.05.

The results of the transcriptome showed that a large proportion of *OfCCCH* genes were differentially expressed in a stress-dependent manner. By comparing the three treatment groups with their control, 4, 5, and 13 DEGs of *OfCCCH* were found under the ABA, salt, and drought stress treatment, respectively (Table S3). After 12 h ABA stress, three genes (*OfCCCH23*, *40*, and *49*) were downregulated, except *OfCCCH32* (Figure 9A). The expression of *OfCCCH7*, *23*, and *32* displayed significant upregulation after salt treatment, while dramatically decreased expression was observed for *OfCCCH49* and *64* (Figure 9A). The expression of *OfCCCH8*, *17*, *18*, *28*, *39*, *45*, and *62* was dramatically stimulated, while significant downregulation was observed for six genes (*OfCCCH2*, *23*, *24*, *27*, *30*, and *60*) under drought stress treatment (Figure 9A). The patterns of expression from 0 h to 24 h of the 16 DEGs of *OfCCCH* were revalidated under different types of abiotic stress using the qRT-PCR method (Figure 9B). The results showed that all the *OfCCCHs* showed obvious abiotic stresses response and were consistent with the patterns of expression of transcriptome. In the ABA stress group, *OfCCCH23*, *32,* and *40* were significantly upregulated at 9 h of treatment and then sharply downregulated after 12 h, and the expression of *OfCCCH49* displayed a significant downregulation after 3 h ABA stress treatment (Figure 9B). In the salt group, *OfCCCH23* and *32* had a similar pattern of expression. They were all highly expressed at 3 h and 9 h and downregulated at 6 h and 24 h, and *OfCCCH49* and *64* were significantly downregulated after 3 h salt stress treatment (Figure 9B). As shown in Figure 9B, six *OfCCCH* genes (*OfCCCH8*, *17*, *23*, *27*, and *28*) significantly changed after 3 h of drought stress treatment, while significant upregulation was observed for *CCCH39*, *45*, and *62* after 6 h of drought stress treatment.

### 2.7. Characterization of the OfCCCH Proteins

**Figure 10:**
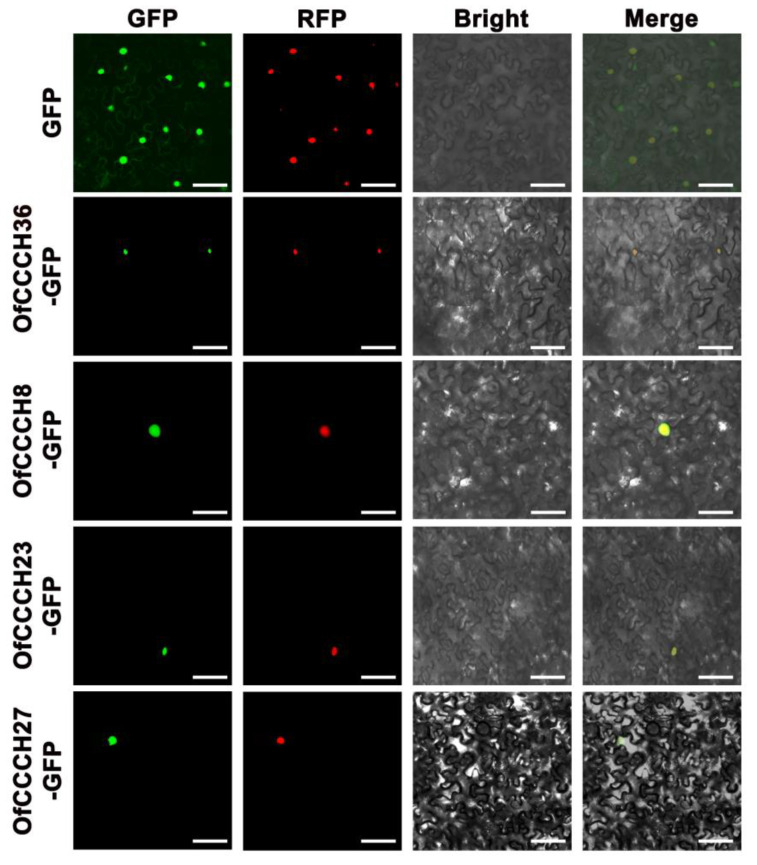
Subcellular localization of OfCCCH8/23/27/36 proteins. Green Fluorescent Protein (GFP) fluorescence signals were observed in the epidermal cells of tobacco leaves. The nucleus was marked by Red Fluorescent Protein (RFP). Bar = 60 µm.

**Figure 11:**
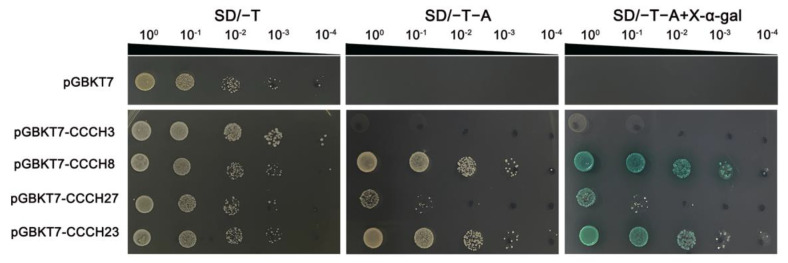
Transcriptional activation activity of OfCCCH8/23/27/36 proteins.

To explore the potential function of the *OfCCCH* genes in the transcriptional regulation system, the representative genes *OfCCCH8*, *23*, *27*, and *36* were fused to a C-terminal GFP and expressed in the leaf epidermis of *N. benthamiana* to detect the subcellular localization. GFP signals were then observed after 36 h. As shown in Figure 10, the GFP fluorescence of the *35S::GFP*-*OfCCCH8*/*23*/*27*/*36* fusion protein was only detected in the nucleus where it could be involved in regulating transcriptional events. To identify whether these *OfCCCH* proteins had trans-activation activity, the proteins (*OfCCCH8*, *23*, *27*, and *36*) were fused in pGBKT7 to form the BD-CCCH vector and then transformed into the AH109 yeast strain. All the yeast cells grew well on the SD/-Trp media. Moreover, *OfCCCH8*, *27*, and *36* grew normally and displayed positive GAL4 activity on X-α-gal-supplemented medium, which suggested that they exhibited transcriptional activation in yeast (Figure 11). However, *OfCCCH23* barely grew on the SD/-T/-A plate, which indicated that *OfCCCH23* lacked transactivation activity in yeast and needed to form a complex with other proteins to perform its function of transcriptional activation (Figure 11).

## 3. Discussion

### 3.1. Characteristic Analysis of OfCCCH TFs in Osmanthus Fragrans

Zinc-finger TFs, as one of the largest TF families in plants, are critical regulators for multiple biological processes, such as morphogenesis, signal transduction, and environmental stress responses [5]. Plants have adopted most typical classes of zinc fingers as functional domains in transcription factors whose functions are manifold and reported in many plants, such as *Arabidopsis* [2], rice [2], and *Populus* [5]. In this study, a total of 66 *CCCH* genes were identified in *O. fragrans* using comprehensive genome-wide analysis (Table S1). Compared with woody plants, the number of *OfCCCHs* was more than that in Longan [6] and less than that in *Populus* [5]. Chromosomal mapping of *OfCCCHs* revealed that the *OfCCCHs* were not uniformly distributed on 21 chromosomes, of which chromosome 3 harbored the most *OfCCCH* numbers (Figure 1). Similarity with *Arabidopsis*, all the *OfCCCH* genes were clustered in nine clades, according to phylogenetic analysis (Figure 2). Previous studies have shown that segmental duplication is largely responsible for the expansion of *Arabidopsis* and rice CCCH gene families [2]. Meanwhile, there were 17 *OfCCCH* gene pairs associated with gene duplication events (Figure 3, Table 1). We speculated that the multiple nature of segmental duplication might contribute to CCCH family expansion in *O. fragrans*. For the gene structure and domain analysis, similar distribution patterns of exons were identified in the same branch of phylogenetic tree (Figure 4), implying that these CCCH subfamily genes likely share a common evolutionary origin and similar biological functions. Previous studies have indicated that CCCH proteins have one to six copies of CCCH-type motifs in animals or plants [6,8,20], while we found that one to seven copies of CCCH-type motifs were contained in *O. fragrans* (Figure 5). In addition, other four-type motifs were identified in the OfCCCH protein sequences, including ANK, KH, PRM, and WD40 motifs. All of these motifs play important roles in diverse molecular processes [21], splicing and transcriptional regulation [22], growth processes [23], and stress response [24]. Many CREs associated with plant hormones were predicted in the *OfCCCH* promoters in *O. fragrans* (Figure 6). It has been reported that CCCHs are involved in responding to hormone signals to regulate plant development and adaptive ability in *Arabidopsis* [25] and switchgrass [15]. Therefore, the results suggested that *OfCCCH* genes might play important roles in the processes of growth and development and stress tolerance by responding of hormone signals in *O. fragrans*.

### 3.2. OfCCCHs Involved in Flower Opening and Abiotic Stress Tolerance in Osmanthus fragrans

Transcriptome analysis is a valuable tool to uncover gene expression profiles in many biological processes of plants. We investigated the expression profiles of *OfCCCH* genes in different tissues and found that all the genes were expressed widely in the tissues (Figure 7). Among them, eight *OfCCCHs* (*OfCCCH3*, *7*, *28*, *32*, *35*, *36*, *40*, and *56*) showed the highest expression in the different tissues of *O. fragrans* (Figure 7), indicating that these *OfCCCHs* might specially regulate the processes of growth and development in these tissues of *O. fragrans*. In addition, *CCCH* genes are extensively involved in the regulation of flowering processes in many plants, such as floral development, flowering time, and flower opening. In *Arabidopsis*, AtKHZ1/2 (CCCH zinc-finger and K-homolog protein) and AtKHZ2 reportedly delay flowering processes [26], while a CCCH Zinc finger protein AaZFP3 from *Adonis amurensis* can promote flowering process in transgenic *A. thaliana* [27]. In the process of flower development, *HUA1* regulates stamen and carpel formation in *A. thaliana* [10]. Additionally, *DR14* encodes a CCCH zinc finger protein that affects the flower opening process in *Ipomoea nil* [28]. During the flower opening processes of *O. fragrans*, a total of 22 *OfCCCHs* (including 9 upregulation and 13 downregulation) were identified and exhibited differential expression from the S1 to the S2 stages (Figure 8, Table S2). The results suggested that these DEGs might be involved in the regulation of flower opening. Particularly, *OfCCCH3*, *16*, *22*, and *36* upregulated more than 5 times at the S2 stage; therefore, these genes might play an important role in flowering initiation. Moreover, *CCCH* genes reportedly respond to various abiotic stresses. For example, *AtZFP1* (*Zinc finger protein 1*) improves salt resistance in *Arabidopsis* [29]. In rice, *OsCCCH10* enhances drought tolerance by regulating stress-related genes [30]. In *O. fragrans*, the differential expression of *OfCCCH* genes were explored after salt, drought, and ABA treatment, and 5, 13, and 4 DEGs were obtained, respectively (Figure 9, Table S3). We found these DEGs had ABA responsiveness CREs in their promoters, suggesting that *OfCCCHs* regulate abiotic tolerance might be associated with the ABA signal in *O. fragrans*. Remarkably, the gene *OfCCCH23* was significantly induced by salt, drought, and ABA at same time. The overlapping responses of *OfCCCH* genes to multiple abiotic stresses provide potential hub players in *O. fragrans* acclimation to stressful conditions. Finally, these obtained *OfCCCH* DEGs play key roles in the regulation of flowering processes and abiotic stresses; therefore, four *OfCCCHs* were selected, including *OfCCCH8*, *23*, *27,* and *36,* to further analyze their subcellular localization and transactivation activity (Figure 10 and Figure 11). The characterization of the four proteins provides important information regarding the investigation of their mechanisms related to the regulation of flowering and abiotic tolerance enhancement in *O. fragrans*.

## 4. Materials and Methods

### 4.1. Plant Materials and Treatments

The materials of *O. fragrans* ‘Yanhong Gui’ are preserved in the Osmanthus Germplasm Resource Garden of Zhejiang Agriculture and Forestry University (Hangzhou, China). All the materials were maintained in a growth chamber (DONGNAN INSTRUMENT Co., Ltd., Ningbo, China) with a 12 h/12 h (light/dark) photoperiod, with a relative humidity of 60%. Samples of five tissues, including root, annual branch, perennial branch, young leaf, and mature leaf, were collected. For the flower opening treatment, six year old uniform plants were sent to the same growth chamber with an ambient temperature of 19 °C. Flower buds with similar sizes at the S1 stage (with the outer bud scales unfurled and the inner bud scales still furled) and the S2 stage (with buds that became globular-shaped and visible inside bracts that covered the inflorescences) were collected. For the abiotic stress treatments, 18 branches of O. *fragrans* were treated with ABA (100 µmol/L), NaCl (200 mmol/L), and mannitol (200 mmol/L) in a growth chamber. In addition, the third or fourth fully expanded leaves from the tip were collected from each plant at 0, 3, 6, 9, 12, and 24 h after treatment. All collected samples were immediately frozen in liquid nitrogen and stored at −80 °C for the subsequent extraction of total RNA and expression profiling. Three biological replicates were established for each treatment.

### 4.2. Genome-Wide Identification of the CCCHs

The genome and protein sequences were obtained from *O. fragrans* genome database to identify the *CCCH* gene family in *O. fragrans* [31]. Information about the *OfCCCH* structural domain (PF00642, https://pfam.xfam.org/family/PF00642, accessed on 10 June 2022) was obtained from the Pfam protein families database (http://pfam.xfam.org/, accessed on 10 June 2022), and HMMER v3.3.2 (http://hmmer.org/, accessed on 10 June 2022) software was used to search all the protein sequences across the genome with a default *e*-value and identify genes with the specific conserved domain. Subsequently, the number of domains present in the protein sequences were validated with two online domain analytical tools, including the Batch Web CD-Search of NCBI (https://www.ncbi.nlm.nih.gov, accessed on 10 June 2022) and SMART (http://smart.embl-heidelberg.de/, accessed on 10 June 2022). 

### 4.3. Chromosomal Localization, Gene Duplication, and Synteny Analysis

The chromosome information of *OfCCCHs* was obtained using TBtools software v1.09 with the GFF3 file, which includes the chromosome length and the start and end sites of genes. MG2C software v2.1 (http://mg2c.iask.in/mg2c_v2.1/, accessed on 12 June 2022) was used to map the chromosomes and the gene locations of the *OfCCCH* gene family. The pattern of duplication pattern of each *OfCCCH* gene was analyzed using MCScanX v1.0 [32]. The Ks (synonymous) and Ka (non-synonymous) substitution ratios of the gene pairs were assessed using DnaSP v5.0 software [33]. A synteny analysis of *OfCCCHs* was conducted using the Quick MCScanX Wrapper program in TBtools. The graph of collinearity analysis was drawn using TBtools software.

### 4.4. Phylogenetic Tree Construction and Sequence Analysis of CCCHs

The amino acid sequences of the CCCH proteins of *O. fragrans* and *Arabidopsis thaliana* were multiplied and aligned using ClustalX [34] and manually corrected. In addition, the phylogenetic tree was constructed using MEGA 11 software [35] and the neighbor-joining method with a bootstrap of 1000. Subsequently, the phylogenetic tree was created using iTOL.v6 software (https://itol.embl.de/, accessed on 12 June 2022). The structures of *OfCCCHs* were visualized using GSDS2.0 (http://gsds.gao-lab.org/, accessed on 12 June 2022). The conserved protein motif of each putative *OfCCCH* family member was analyzed using the MEME program with the motif number to be identified established at 20 and the other parameters set as default. Finally, the information on the molecular weight and the isoelectric points of *OfCCCHs* were obtained using the online tool EXPASy Compute pI/Mw (https://web.expasy.org/compute_pi/, accessed on 12 June 2022).

### 4.5. Gene Structure, Domain, and CREs (Cis-Regulatory Elements) Prediction for the OfCCCHs

The gene structures of the *OfCCCHs* were predicted using Gene Structure Display Server 2.0 (http://gsds.cbi.pku.edu.cn/, accessed on 1 July 2022). The domains of *OfCCCHs* were analyzed using the NCBI CD-Search program (https://www.ncbi.nlm.nih.gov/Structure/cdd/wrpsb.cgi, accessed on 1 July 2022) by sending *OfCCCH* protein sequences that had been visualized to the TBtools of Visualize NCBI CDD Domain pattern program. CREs in the *OfCCCHs* promoters, which were defined as 2000 bp genomic regions upstream of translational start codons, were detected with two online tools, Plantcare (http://bioinformatics.psb.ugent.be/webtools/plantcare/html/, accessed on 1 July 2022) and NewPLACE (https://www.dna.affrc.go.jp/PLACE/?action=newplace, accessed on 1 July 2022).

### 4.6. Total RNA Extraction and RNA-Sequencing

The total RNA was extracted using an RNAprep pure Kit (TianGen Biotech Co., Ltd., Beijing, China) following the manufacturer’s instructions. A 3 µg pool of RNA was used for transcriptome sequencing with three biological replicates using the Illumina Hiseq 2500 platform. Clean reads were obtained by removing reads that contained adapters and poly-N and low-quality reads from the raw data. Unigenes were aligned using BLASTx (*e*-value < 1 × 10^−5^) against NR (www.ncbi.nlm.nih.gov/refseq, accessed on 11 January 2022), Swiss-Prot (www.uniprot.org, accessed on 11 January 2022), GO (http://geneontology.org/, accessed on 11 January 2022), and KEGG (https://www.genome.jp/kegg/, accessed on 11 January 2022) databases. FeatureCounts v1.5.0-p3 was used to count the reads numbers mapped to each gene. Additionally, FPKM (the fragments per kilobase of exon per million mapped reads) was calculated based on the length of the gene and the reads count mapped to this gene. Differential expression between the two conditions was analyzed using the DESeq2 method. Genes with |log2FC| > 1 and an adjusted *p*-value ≤ 0.05 found using the DESeq2 method were assigned as differentially expressed genes.

### 4.7. Quantitative Real-Time PCR Analysis

The cDNA synthesis was completed using HiScript II Q Select RT Supermix for qPCR (Vazyme Biotech Co., Ltd., Nanjing, China). The qRT-PCR reactions were performed using the LightCycler480II System (Roche, Basel, Switzerland) as described by Yang et al. [36]. The *OfACT* gene was used as internal normalization for *O. fragrans* [37]. Triple biological replications were performed for each treatment. The relative level of expression of the target gene was calculated using the 2^−∆∆CT^ method. The primers were designed using primer 5.0 software, primer sequences are showed in Table S4.

### 4.8. Subcellular Localization of the OfCCCH Proteins

The sequences of ORF (open reading frame) without the termination codon were amplified, and then PCR products were subcloned into the pORE R4 vector (genbank: ay562547.1) as an in-frame C-terminal fusion with GFP (green fluorescent protein). The resulting construct was confirmed via sequencing and transformed into the *Agrobacterium tumefaciensstrain* strain GV3101 (Weidi Biotechnology Co., Ltd., Shanghai, China). The transformed *A. tumefaciens* lines were infiltrated into fully expanded leaves of tobacco plants, and each experiment was repeated twice. The GFP signal was investigated using a Zeiss LSM 710 (Carl Zeiss, Jena, Germany) after 36 h. All primer information was showed in the Table S4.

### 4.9. Transcriptional Activation Analysis

A yeast two-hybrid system was used to analyze the transcriptional activation of the *OfCCCHs*. The full-length sequences of *OfCCCH* proteins were cloned and fused in the pGBKT7-BD vector. The empty vector was used as a negative control. The vectors were then transformed into the *Saccharomyces cerevisiae* strain AH109 which contained a MEL1 reporter that encoded α-galactosidase. The expressed MEL1 reporter gene could be detected if the recombinant vector pGBKT7-*OfCCCHs* was transactivated. SD/-Trp media was used to screen for positive transformants, and the positive clones were further screened on SD/-Trp/-Ade and incubated at 30 °C for 3 days.

## 5. Conclusions

In this study, a total of 66 *OfCCCH* genes were identified in *O. fragrans* using whole genome analysis. Transcriptome and qRT-PCR analysis results identified 22 differently expressed genes in the flower opening stages, and 5, 13, and 4 differently expressed genes were obtained after salt, drought, and ABA treatment, respectively. The comprehensive analysis of expression patterns demonstrated the importance of *OfCCCH* genes in the regulation of flower opening and response to abiotic stresses. 

## Figures and Tables

**Table 1:** Analysis of Ka/Ks and time of divergence estimation for *OfCCCHs*.

Duplicated Gene Pairs	Ka	Ks	Ka/Ks	Type of Duplication	Type of Selection	Divergene Time (Mya)
*CCCH2-CCCH38*	0.371	1.127	0.329	Segmental	Purifying	37.57
*CCCH4-CCCH45*	0.123	0.266	0.461	Segmental	Purifying	8.87
*CCCH3-CCCH54*	0.053	0.311	0.171	Segmental	Purifying	10.37
*CCCH11-CCCH17*	0.050	0.295	0.168	Segmental	Purifying	9.83
*CCCH10-CCCH60*	0.052	0.286	0.181	Segmental	Purifying	9.53
*CCCH8-CCCH59*	0.141	0.243	0.581	Segmental	Purifying	8.11
*CCCH13-CCCH58*	0.066	0.273	0.243	Segmental	Purifying	9.10
*CCCH15-CCCH31*	0.072	0.298	0.241	Segmental	Purifying	9.93
*CCCH19-CCCH65*	0.086	0.237	0.364	Segmental	Purifying	7.91
*CCCH20-CCCH66*	0.166	0.562	0.295	Segmental	Purifying	18.73
*CCCH23-CCCH40*	0.133	0.522	0.256	Segmental	Purifying	17.41
*CCCH26-CCCH39*	0.126	0.258	0.491	Segmental	Purifying	8.62
*CCCH25-CCCH42*	0.103	0.366	0.281	Segmental	Purifying	12.23
*CCCH24-CCCH55*	0.201	0.596	0.338	Segmental	Purifying	19.87
*CCCH36-CCCH49*	0.061	0.333	0.184	Segmental	Purifying	11.12
*CCCH43-CCCH53*	0.043	0.211	0.201	Segmental	Purifying	7.03
*CCCH58- CCCH61*	0.005	0.011	0.438	Segmental	Purifying	0.37

Note: Ka, non-synonymous; Ks, synonymous.

## Data Availability

Not applicable.

## Supplementary Materials

The following supporting information can be downloaded at: https://www.mdpi.com/article/10.3390/ijms232315363/s1.
