# Supplementary material for: Comparative Transcriptome Analysis of CCCH Family in Roles of Flower Opening and Abiotic Stress in Osmanthus fragrans
Source: Int J Mol Sci. 2022 Dec 6;23(23):15363. doi: 10.3390/ijms232315363 (PMC9735588; doi:10.3390/ijms232315363)
Supplement: Supplementary file 1 [file ijms-23-15363-s001.zip › Table S1.pdf]

Table S1 Characterization of the predicted *CCCH* proteins in *O. fragrans*

Gene name	Number of amino acids	Molecular weight (kDa)	PI	Chr
CCCH1	1024	113.94	7.82	1
CCCH2	388	43.67	6.20	1
CCCH3	331	37.43	7.95	1
CCCH4	477	52.26	8.03	1
CCCH5	785	86.76	6.29	1
CCCH6	407	45.57	5.71	1
CCCH7	396	43.87	8.15	2
CCCH8	406	44.08	8.93	3
CCCH9	451	49.57	6.02	3
CCCH10	375	43.02	5.12	3
CCCH11	742	81.04	6.17	3
CCCH12	394	42.69	6.31	3
CCCH13	299	31.69	9.14	3
CCCH14	799	87.50	5.70	3
CCCH15	330	31.65	9.37	4
CCCH16	337	37.12	6.92	4
CCCH17	667	72.85	6.23	5
CCCH18	667	72.82	6.23	5
CCCH19	732	79.42	5.74	6
CCCH20	345	39.03	8.22	6
CCCH21	443	48.68	8.56	6
CCCH22	446	50.91	5.55	7
CCCH23	320	35.83	9.25	7
CCCH24	690	75.53	5.63	7
CCCH25	325	34.24	9.10	7
CCCH26	767	82.82	4.60	7
CCCH27	339	38.52	6.47	8
CCCH28	367	41.10	7.14	8
CCCH29	788	87.83	8.90	9
CCCH30	1006	112.43	6.49	9
CCCH31	287	30.32	9.23	9
CCCH32	331	36.76	7.07	9
CCCH33	425	46.35	8.10	9
CCCH34	331	36.90	7.67	9
CCCH35	287	31.61	8.84	10
CCCH36	395	42.53	8.14	11
CCCH37	147	16.94	9.28	11
CCCH38	234	25.91	9.47	13
CCCH39	761	81.79	4.54	13
CCCH40	364	40.60	8.44	13
CCCH41	365	40.33	7.61	13
CCCH42	303	31.80	9.13	13

CCCH43	293	30.32	9.03	14
CCCH44	528	59.34	5.24	15
CCCH45	433	47.86	8.42	15
CCCH46	1195	130.48	5.13	15
CCCH47	352	38.28	6.02	16
CCCH48	767	89.36	5.88	16
CCCH49	663	72.09	6.40	16
CCCH50	172	19.37	7.03	16
CCCH51	351	38.83	8.82	17
CCCH52	502	56.37	9.05	17
CCCH53	495	52.19	8.60	17
CCCH54	351	39.66	6.59	18
CCCH55	736	80.88	5.72	18
CCCH56	260	29.63	9.07	18
CCCH57	370	39.34	5.60	19
CCCH58	349	37.38	9.08	21
CCCH59	379	41.31	8.11	21
CCCH60	313	36.05	8.91	21
CCCH61	324	34.71	9.12	21
CCCH62	1008	111.89	6.30	22
CCCH63	685	76.75	6.45	23
CCCH64	748	79.11	5.80	23
CCCH65	695	75.90	6.12	23
CCCH66	260	29.96	9.38	23
