# Supplementary material for: Comparative Transcriptome Analysis of CCCH Family in Roles of Flower Opening and Abiotic Stress in Osmanthus fragrans
Source: Int J Mol Sci. 2022 Dec 6;23(23):15363. doi: 10.3390/ijms232315363 (PMC9735588; doi:10.3390/ijms232315363)
Supplement: Supplementary file 1 [file ijms-23-15363-s001.zip › Table S2.pdf]

Table S2 Expression profiles of *OfCCCH* DEGs at S1 and S2 stages of flower opening

Gene	FPKM		$Log_2FoldChange$	P value
	0 h	120 h		
OfCCCH3	0.09	155.58	9.76	1.1×10^{-14}
OfCCCH6	563.67	1379.78	1.29	3.7×10^{-29}
OfCCCH7	8134.99	100.62	-6.33	1.0×10^{-189}
OfCCCH16	7.18	60.73	3.09	1.8×10^{-10}
OfCCCH22	925.09	451.49	-1.04	2.1×10^{-17}
OfCCCH23	1302.30	112.21	-3.53	2.0×10^{-105}
OfCCCH25	8015.99	2695.55	-1.57	2.3×10^{-69}
OfCCCH27	20.36	44.67	1.13	6.8×10^{-3}
OfCCCH28	373.68	16.23	-4.49	2.9×10^{-48}
OfCCCH35	339.98	108.03	-1.65	1.9×10^{-10}
OfCCCH36	154.18	322.65	1.06	2.2×10^{-4}
OfCCCH38	26.64	9.60	-1.46	7.9×10^{-3}
OfCCCH40	2427.85	51.47	-5.57	2.3×10^{-248}
OfCCCH43	483.78	165.12	-1.55	4.6×10^{-20}
OfCCCH49	772.92	11983.74	3.96	1.0×10^{-89}
OfCCCH53	553.11	255.33	-1.12	1.0×10^{-13}
OfCCCH55	388.68	170.69	-1.19	5.7×10^{-12}
OfCCCH57	3028.77	6458.62	1.09	3.8×10^{-32}
OfCCCH58	142.91	290.19	1.02	8.3×10^{-7}
OfCCCH61	71.53	162.94	1.19	2.5×10^{-6}
OfCCCH63	1501.08	676.35	-1.15	1.4×10^{-14}
OfCCCH64	0.01	9.80	-2.85	7.4×10^{-11}
