# Supplementary material for: Comparative Transcriptome Analysis of CCCH Family in Roles of Flower Opening and Abiotic Stress in Osmanthus fragrans
Source: Int J Mol Sci. 2022 Dec 6;23(23):15363. doi: 10.3390/ijms232315363 (PMC9735588; doi:10.3390/ijms232315363)
Supplement: Supplementary file 1 [file ijms-23-15363-s001.zip › Table S3.pdf]

Table S3 Expression profiles of *OfCCCH* DEGs under different abiotic stresses

Stress	Gene	FPKM		$Log_2FoldChange$	P value
		0 h	12 h		
ABA	*OfCCCH23*	41.29	32.40	-1.60	4.9×10^{-3}
	OfCCCH32	27.53	63.30	1.21	3.3×10^{-3}
	OfCCCH40	0.83	0.15	-2.51	4.8×10^{-2}
	OfCCCH49	90.87	42.41	-1.09	5.3×10^{-9}
Salt	*OfCCCH7*	4.37	10.95	1.33	1.4×10^{-6}
	OfCCCH23	9.36	32.40	1.79	2.2×10^{-23}
	OfCCCH32	27.53	63.61	1.21	2.5×10^{-9}
	OfCCCH49	90.87	31.99	-1.50	1.2×10^{-39}
	OfCCCH64	0.53	0.20	-1.43	4.3×10^{-2}
Drought	*OfCCCH2*	13.00	4.27	-1.59	6.5×10^{-10}
	OfCCCH8	5.22	11.46	1.12	1.9×10^{-5}
	OfCCCH17	14.18	34.83	1.38	1.2×10^{-17}
	OfCCCH18	13.72	36.96	1.26	7.9×10^{-15}
	OfCCCH23	26.64	9.60	-1.46	7.9×10^{-3}
	OfCCCH24	1.58	0.51	-1.63	6.2×10^{-5}
	OfCCCH27	0.36	0.12	-5.72	7.6×10^{-3}
	OfCCCH28	10.95	38.69	1.80	9.8×10^{-16}
	OfCCCH30	19.42	7.19	-1.42	1.2×10^{-22}
	OfCCCH39	17.76	67.56	1.72	6.4×10^{-52}
	OfCCCH45	23.57	45.98	1.07	1.9×10^{-10}
	OfCCCH60	17.83	6.74	-1.39	6.4×10^{-11}
	OfCCCH62	7.19	12.78	1.15	1.8×10^{-12}
