# Supplementary material for: Comparative Transcriptome Analysis of CCCH Family in Roles of Flower Opening and Abiotic Stress in Osmanthus fragrans
Source: Int J Mol Sci. 2022 Dec 6;23(23):15363. doi: 10.3390/ijms232315363 (PMC9735588; doi:10.3390/ijms232315363)
Supplement: Supplementary file 1 [file ijms-23-15363-s001.zip › Table S4.pdf]

Table S4 Primers information of *OfCCCH* genes

Type	Gene	Primer sequence（forward/reverse primer）	Annealing Temperature (℃)
qRT-PCR	*OfACT*	F- CCCAAGGCAAACAGAGAAAAAAT,<br>R- ACCCCATCACCAGAATCAAGAA	60.0
	OfCCCH6	F- TTCGGGAGTGCGTGTCTTTT,<br>R- CTTTGAGGCATGCCAATCCG	59.5
	OfCCCH7	F- GGTCACATGACTGGACGGAG,<br>R- CCCTTTCTGAAATCCGGGCA	58.6
	OfCCCH8	F- TTCATATGTGCCCGGTGCTT,<br>R- CAGGGCTAACTGGTCCCAAG	60.3
	OfCCCH17	F- GATCGTGGTCATCGGTCGAG,<br>R- CCCATTGGCATCAACAGCAC	61.2
	OfCCCH23	F- TCTTTGCTCACTCGACGGAC,<br>R- CAATCGGTGGAGAACCCGAA	58.9
	OfCCCH25	F- GCCAGTTGTGAAGGCTTGTG,<br>R- GTGGCAACTCTCGCCAAATG	60.6
	OfCCCH27	F- CTCGGGGGCTAAAGGAACAA,<br>R- CAAATGTGCAATGCTCCCCA	60.5
	OfCCCH28	F- AGATGTTCCGAACCCGAACC,<br>R- CGAGATCCACTCCACATCCG	59.7
	OfCCCH32	F- CATGTGGCTCAGATGGGGTT,<br>R- GGCCGAGGCATCGATTAAGA	59.7
	OfCCCH36	F- GCTGCAGCATTGAGCCTTTT,<br>R- GGGACATGGGCTGGTTGTAT	59.6
	OfCCCH39	F- GCAGCTGCGTAAGTGGAGTA,<br>R- TGTCCGCTTAAAGCATCCGT	60.0
	OfCCCH40	F- AAGGGTGACGCGTGTGAATA,<br>R- TTGCATGGCTGCGTACGATA	59.4
	OfCCCH45	F- TCCCAACAGGAGGTCCTAGC,<br>R- GGTCTGCCGACTGTGGATAC	59.5
	OfCCCH49	F- GGCTCCGCTCTTTCCTTTGA,<br>R- TCACATCAACTGGGCGATGA	60.5
	OfCCCH57	F- CCCACCCATTCATACGGACC,<br>R- AGAGTGTTTCAGCGGAAGGG	60.3
	OfCCCH62	F- AGAAACGGAAAAGGGGACGG,<br>R- CACGAGACAACCCCCATCAA	60.1
	OfCCCH64	F- TTCGGCTGCTTCTAGTCCAG,<br>R- TTGACTCGGGCGTTTGATGA	60.8
pORE R4 vector	*OfCCCH8*	F- TTGGGGCCCAACGTTCTCGAGATGGAGTTGTACGGAAGGAGAAGC<br>R- CGAATTCTCCCGGGTGGATCCAAACAGGTTGAAAACATCAGATGAACA	60.6
	OfCCCH23	F- TTGGGGCCCAACGTTCTCGAGATGGGTAGTATTTGTGCTGAACAGC<br>R- CGAATTCTCCCGGGTGGATCCAATTAGGTTAATAGATCATTGACCCACCC	61.9
	OfCCCH27	F- TTGGGGCCCAACGTTCTCGAGATGAGTTTCCCTGATCCTGCAC<br>R- CGAATTCTCCCGGGTGGATCCAATATATACTCTTTCCTAGGGGAGTTGTGT	60.5
	OfCCCH36	F- TTGGGGCCCAACGTTCTCGAGATGTGCAGTGGCCCAGAGAA<br>R- CGAATTCTCCCGGGTGGATCCAAGGAGGGAGATGAAGGGATGG	60.9
pGBKT7 vector	*OfCCCH8*	F- ATGGCCATGGAGGCCGAATTCATGGAGTTGTACGGAAGGAGAAGC<br>R- CCGCTGCAGGTCGACGGATCCTTAACAGGTTGAAAACATCAGATGAA	61.5
	OfCCCH23	F- ATGGCCATGGAGGCCGAATTC ATGGGTAGTATTTGTGCTGAACAGC<br>R- CCGCTGCAGGTCGACGGATCC TTAGGTTAATAGATCATTGACCCACC	61.2
	OfCCCH27	F- ATGGCCATGGAGGCCGAATTC ATGAGTTTCCCTGATCCTGCAC<br>R- CCGCTGCAGGTCGACGGATCC TCATATATACTCTTTCCTAGGGGAGTTG	60.6
	OfCCCH36	F- ATGGCCATGGAGGCCGAATTC ATGTGCAGTGGCCCAGAGAA<br>R- CCGCTGCAGGTCGACGGATCC CTAGGAGGGAGATGAAGGGATGG	62.1
